# A Missed Clue in Plain Sight: Hyperproteinemia and Nonlytic Skeletal Disease Revealing Immunoglobulin A Multiple Myeloma

**DOI:** 10.7759/cureus.108969

**Published:** 2026-05-16

**Authors:** Alireza Izadian Bidgoli, Alberto Gomez Veliz

**Affiliations:** 1 Medicine, American University of the Caribbean School of Medicine, Cupecoy, SXM; 2 Internal Medicine, Jackson Memorial Hospital, Miami, USA

**Keywords:** diffuse osteopenia, flow cytometry, high-risk cytogenetics, iga multiple myeloma, vertebral compression fractures

## Abstract

IgA multiple myeloma can present with atypical and misleading clinical features, delaying diagnosis when more common conditions dominate the initial presentation. We report the case of a 50-year-old male with a history of recurrent gastrointestinal bleeding who presented with severe symptomatic anemia requiring transfusion. Initial evaluation focused on suspected acute blood loss; however, persistent cytopenias and markedly elevated total protein with a preserved albumin level raised concern for an underlying systemic process. Further laboratory evaluation revealed a significant globulin gap, with total protein levels exceeding 10 g/dL and markedly elevated immunoglobulin A (IgA) at 6,318 mg/dL. Serum free light chain analysis demonstrated elevated kappa chains with a profoundly abnormal kappa/lambda ratio. Imaging studies identified multiple vertebral compression fractures and diffuse osteopenia, while endoscopic evaluation revealed severe esophagitis without active bleeding to fully account for the degree of anemia. Bone marrow biopsy confirmed clonal plasma cell proliferation consistent with IgA multiple myeloma. This case underscores the diagnostic importance of recognizing hyperproteinemia and the globulin gap as early indicators of plasma cell dyscrasia, particularly in patients with competing clinical diagnoses such as gastrointestinal bleeding. It further highlights how multiple myeloma may present without classic features such as renal dysfunction or hypercalcemia, instead manifesting through indirect findings, including skeletal fragility and unexplained cytopenias. Early recognition of these subtle but critical clues can facilitate timely diagnosis and initiation of therapy, ultimately improving patient outcomes.

## Introduction

Multiple myeloma is a clonal plasma cell malignancy characterized by monoclonal protein production and end-organ damage, classically defined by hypercalcemia, renal dysfunction, anemia, and bone disease (CRAB (calcium elevation, renal dysfunction, anemia, and bone lesions) criteria) [1]. Despite well-established diagnostic frameworks, the clinical presentation of multiple myeloma remains highly heterogeneous, and atypical manifestations frequently delay diagnosis [2]. Among subtypes, IgA multiple myeloma accounts for approximately 20% of cases and is often associated with higher tumor burden, elevated serum viscosity, and less readily detectable monoclonal spikes on routine electrophoresis, contributing to diagnostic challenges [3].

Hyperproteinemia and the “globulin gap,” defined as the difference between total protein and albumin, represent underrecognized but important biochemical clues suggestive of monoclonal gammopathy [4]. In patients presenting with competing diagnoses, such as gastrointestinal bleeding, these findings may be overlooked, leading to anchoring bias and delayed recognition of underlying plasma cell dyscrasia. Furthermore, skeletal manifestations of myeloma may initially be misattributed to osteoporosis or degenerative disease, particularly in younger patients or those with prior vertebral pathology [5].

We present a case of IgA multiple myeloma in which recurrent gastrointestinal bleeding and severe anemia obscured the underlying diagnosis. This case highlights the critical importance of recognizing hyperproteinemia and subtle laboratory abnormalities as early indicators of plasma cell malignancy, even in the absence of classic CRAB features. This case uniquely demonstrates how a globulin gap can serve as the earliest diagnostic pivot in IgA myeloma masked by gastrointestinal bleeding, an underrecognized clinical scenario not well described in existing literature.

## Case presentation

Clinical presentation

A 50-year-old male with a history of alcohol use disorder, bipolar disorder, hepatic steatosis, recurrent upper gastrointestinal bleeding, chronic back pain, and multiple prior vertebral compression fractures status post kyphoplasty at T7, T10, L1, and L4 presented with worsening back pain and dark stools. He reported several days of severe back pain that worsened after sneezing, without recent trauma, focal weakness, saddle anesthesia, or bowel or bladder dysfunction. He also reported dark bowel movements concerning recurrent gastrointestinal bleeding. His prior history was notable for multiple admissions for upper gastrointestinal bleeding requiring transfusion support, as well as known esophagitis, esophageal ulceration, and a large hiatal hernia.

Physical examination and initial assessment

On presentation, the patient was hemodynamically stable and in no acute distress. He was alert and oriented, with no focal neurologic deficits. Cardiopulmonary examination was unremarkable, and abdominal examination revealed a soft, nondistended abdomen without tenderness, rebound, or guarding. Musculoskeletal examination was notable for thoracic back pain without focal motor or sensory deficits. Orthopedic evaluation documented intact strength in the bilateral upper and lower extremities, intact sensation, and no clonus. Given the combination of severe anemia, recurrent gastrointestinal bleeding, thrombocytopenia, persistent vertebral fractures, and markedly abnormal protein studies, the initial assessment expanded beyond acute blood loss anemia and raised concern for an underlying plasma cell dyscrasia.

Laboratory results and specialized studies

Initial laboratory evaluation revealed profound hematologic abnormalities, most notably severe anemia with hemoglobin levels as low as 5.4-5.5 g/dL on admission, necessitating multiple packed red blood cell transfusions, with subsequent stabilization to approximately 9.0-9.4 g/dL. This was accompanied by persistent thrombocytopenia, with platelet counts ranging from 82 to 106 × 10³/µL, and intermittent leukopenia, with white blood cell counts between 3.3 and 5.3 × 10³/µL. Red cell distribution width was markedly elevated (>20%), and peripheral smear demonstrated significant anisopoikilocytosis, including microcytosis, macrocytosis, hypochromia, polychromasia, and large platelets. Collectively, these findings suggested a complex bone marrow process beyond isolated blood loss or nutritional deficiency (Table 1).

Serum chemistry was notable for marked hyperproteinemia, with total protein levels reaching 10.4-10.5 g/dL in the setting of only mildly reduced albumin (3.6-3.8 g/dL), resulting in a pronounced globulin gap. Subsequent evaluation for plasma cell dyscrasia demonstrated markedly elevated IgA (6,318 mg/dL), significantly increased kappa free light chains (119.45 mg/L), and a profoundly abnormal kappa/lambda ratio of 663.61. The gamma globulin fraction was elevated at 4.7 g/dL, further supporting the presence of a monoclonal gammopathy. Renal function remained preserved throughout hospitalization, with creatinine ranging from 0.7 to 0.9 mg/dL and estimated glomerular filtration rate exceeding 90 mL/min/1.73 m². Serum calcium levels remained within normal limits. Liver-associated enzymes demonstrated mild transaminitis and cholestatic elevation, including AST up to 88 U/L, ALT up to 119 U/L, and alkaline phosphatase up to 172 U/L. Coagulation studies revealed mild prolongation of prothrombin time, with INR ranging from 1.15 to 1.25 (Table 1).

**Table 1:** Summary of hematologic, biochemical, and plasma cell dyscrasia related laboratory findings during hospitalization. Key laboratory findings at admission and throughout hospitalization, highlighting severe anemia requiring transfusion, persistent thrombocytopenia, intermittent leukopenia, and marked hyperproteinemia with a significant globulin gap. Specialized studies demonstrate a monoclonal IgA paraproteinemia with markedly elevated IgA levels, increased kappa free light chains, and a profoundly abnormal kappa/lambda ratio, supporting the diagnosis of IgA multiple myeloma. Renal function remained preserved, and no hypercalcemia was observed. Mild transaminitis and cholestatic enzyme elevation were present, along with a mild prolongation of coagulation parameters. WBC: white blood cell count; RDW: red cell distribution width; IgA: immunoglobulin A; eGFR: estimated glomerular filtration rate; AST: aspartate aminotransferase; ALT: alanine aminotransferase; INR: international normalized ratio.

Category	Parameter	At Admission	Range During Hospitalization	Reference Range (Unit)	Interpretation
Hematology	Hemoglobin	5.4-5.5	5.4-9.4	13.5-17.5 g/dL	Severe anemia, transfusion responsive
	Platelets	82	82-106	150-400 × 10³/µL	Persistent thrombocytopenia
	WBC	3.3	3.3–5.3	4.0-11.0 × 10³/µL	Intermittent leukopenia
	RDW	>20	>20	11.5–14.5%	Markedly elevated, anisocytosis
	Peripheral smear	—	Mixed abnormalities	—	Suggestive of marrow/infiltrative process
Chemistry	Total protein	10.5	10.4–10.5	6.0–8.3 g/dL	Marked hyperproteinemia
	Albumin	3.8	3.6–3.8	3.5–5.0 g/dL	Mildly decreased
	Globulin gap	Elevated	Elevated	<4 g/dL	Suggestive of monoclonal protein
Plasma cell workup	IgA	—	6,318	70–400 mg/dL	Markedly elevated, monoclonal
	Kappa free light chains	—	119.45	3.3–19.4 mg/L	Elevated
	Kappa/Lambda ratio	—	663.61	0.26–1.65	Profoundly abnormal
	Gamma globulin	—	4.7	0.7–1.5 g/dL	Elevated
Renal	Creatinine	0.7	0.7–0.9	0.6–1.3 mg/dL	Preserved renal function
	eGFR	>90	>90	>60 mL/min/1.73 m²	Normal
Electrolytes	Calcium	Normal	Normal	8.5–10.5 mg/dL	No hypercalcemia
Liver	AST	—	up to 88	10-40 U/L	Elevated
	ALT	—	up to 119	7-56 U/L	Elevated
	Alkaline phosphatase	—	up to 172	44-147 U/L	Elevated
Coagulation	INR	1.15	1.15-1.25	0.8-1.1	Mild prolongation

**Figure 1:**
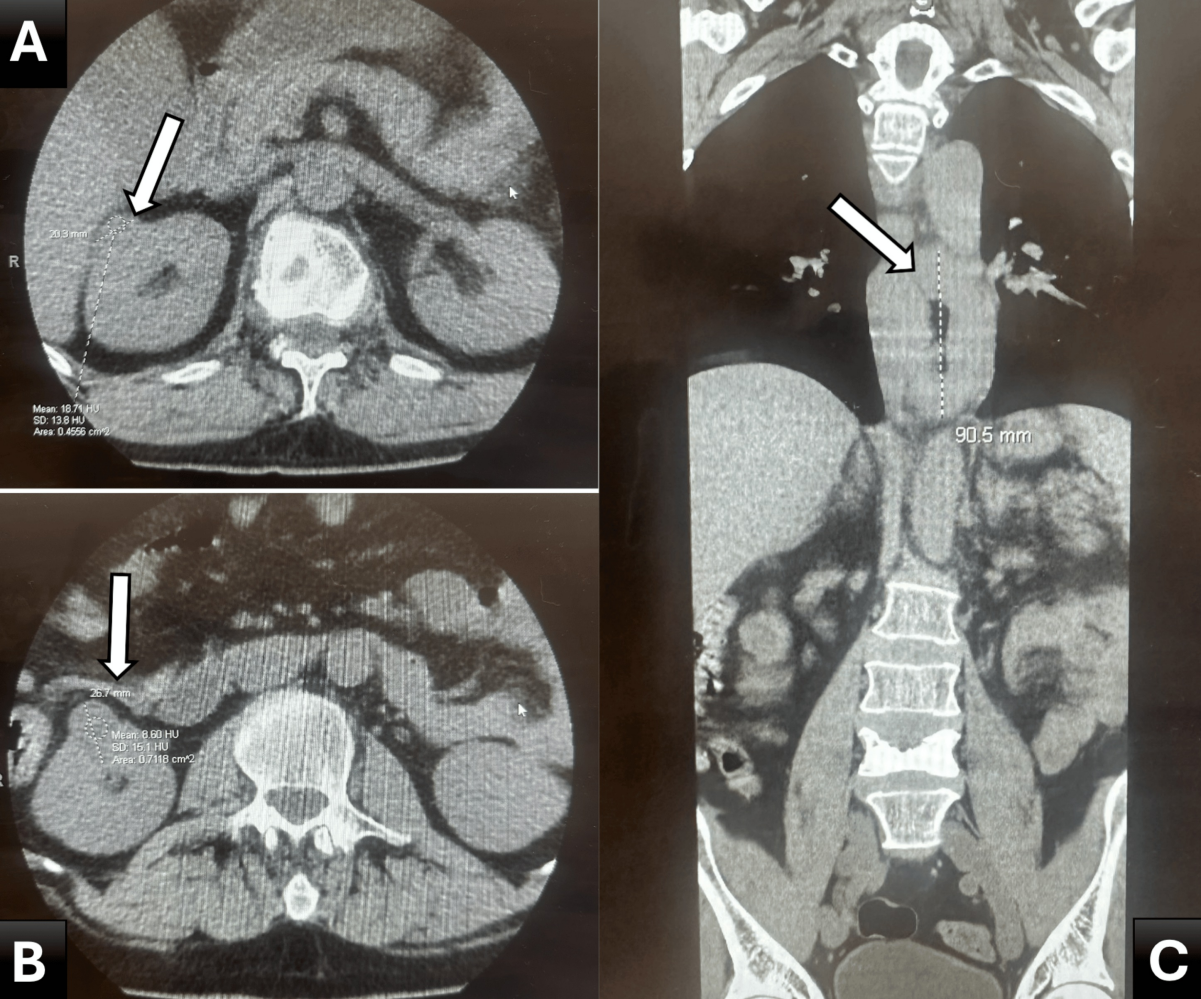
CT imaging demonstrating esophageal wall thickening in the absence of focal osteolytic lesions. (A, B) Axial CT images demonstrating circumferential thickening of the distal esophageal wall (arrows), measuring approximately 20–27 mm. (C) Coronal CT reconstruction showing longitudinal esophageal wall thickening measuring approximately 9 cm. Notably, no discrete osteolytic lesions are identified on these images despite the presence of extensive skeletal involvement elsewhere, illustrating an atypical radiographic presentation of multiple myeloma characterized by diffuse marrow infiltration rather than focal bone destruction.

Cross-sectional imaging revealed additional systemic findings, including circumferential wall thickening of the mid-to-distal esophagus measuring approximately 9 cm in length, raising concern for a concurrent pathologic process and warranting further clinical correlation (Figure 1).

**Figure 2:**
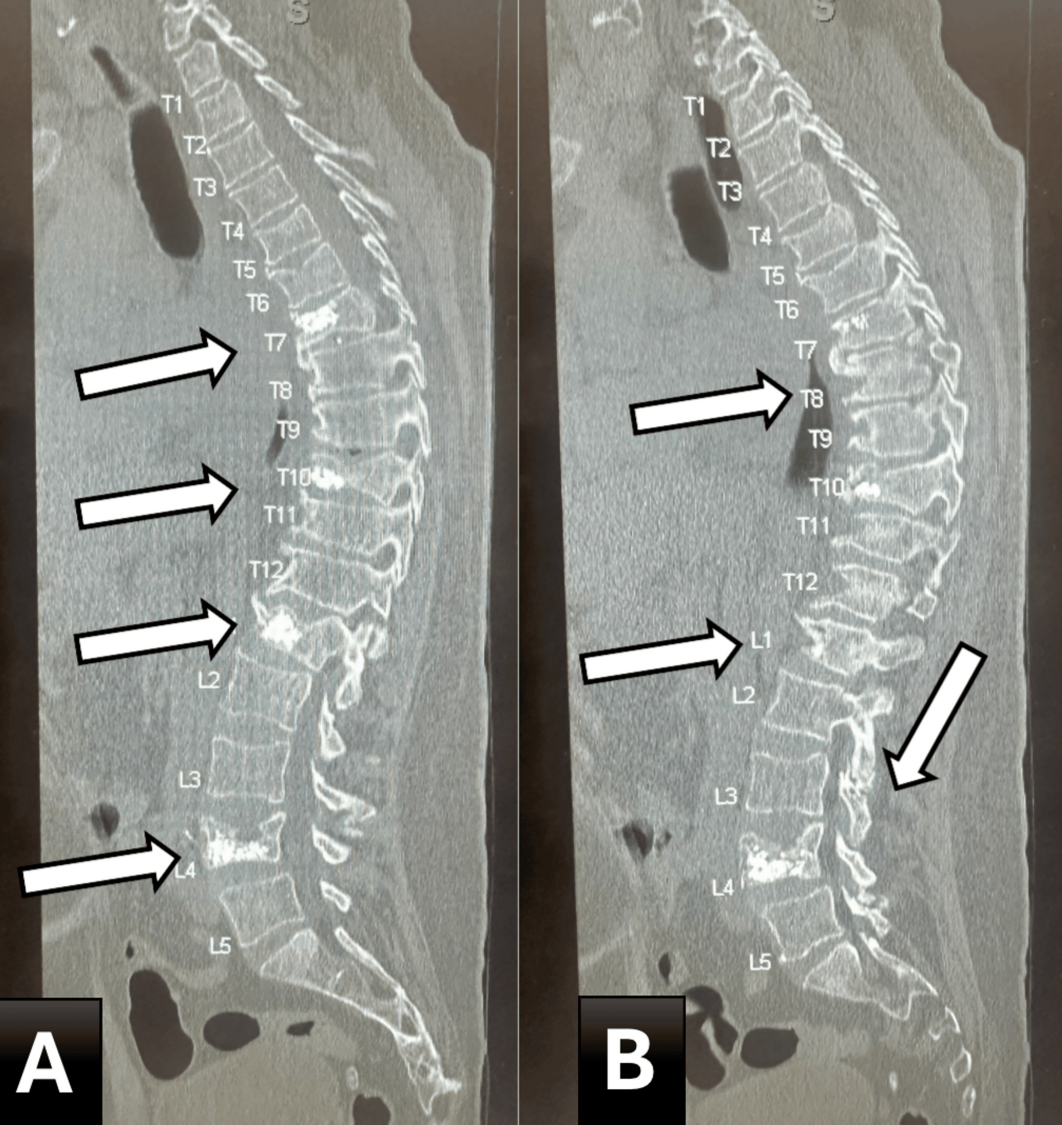
Multilevel vertebral compression fractures in the absence of focal osteolytic lesions. (A, B) Sagittal CT reconstructions of the thoracic and lumbar spine demonstrating multiple vertebral compression deformities (arrows), involving levels T7, T8–T11, and L1–L4. Several vertebral bodies demonstrate post-kyphoplasty changes with hyperdense cement material. Despite extensive structural compromise and multilevel involvement, no discrete osteolytic lesions are identified, further supporting an atypical radiographic presentation of multiple myeloma characterized by diffuse marrow infiltration rather than focal bone destruction.

Advanced skeletal imaging demonstrated extensive, multifocal involvement. Computed tomography (CT) of the thoracic and lumbar spine revealed diffuse osteopenia with multiple chronic compression deformities involving the T5, T7, T10, T11, T12, L1, and L4 vertebral bodies, several of which had undergone prior kyphoplasty (Figure 2). A new acute compression fracture of the T6 vertebral body was identified, involving both the superior endplate and the posterior vertebral body, with associated paravertebral soft-tissue density suggestive of an adjacent hematoma, likely secondary to acute vertebral collapse and osseous fragility in the setting of diffuse marrow infiltration and thrombocytopenia, without evidence of retropulsion. Serial imaging further demonstrated progressive skeletal fragility with recurrent fractures despite prior vertebral augmentation, in addition to diffuse degenerative changes, including spondylosis and facet hypertrophy. Notably, despite the extent of skeletal involvement, no discrete lytic lesions were identified, highlighting an atypical radiographic pattern characterized by diffuse osteopenia and marrow infiltration rather than classic focal osteolysis.

**Figure 3:**
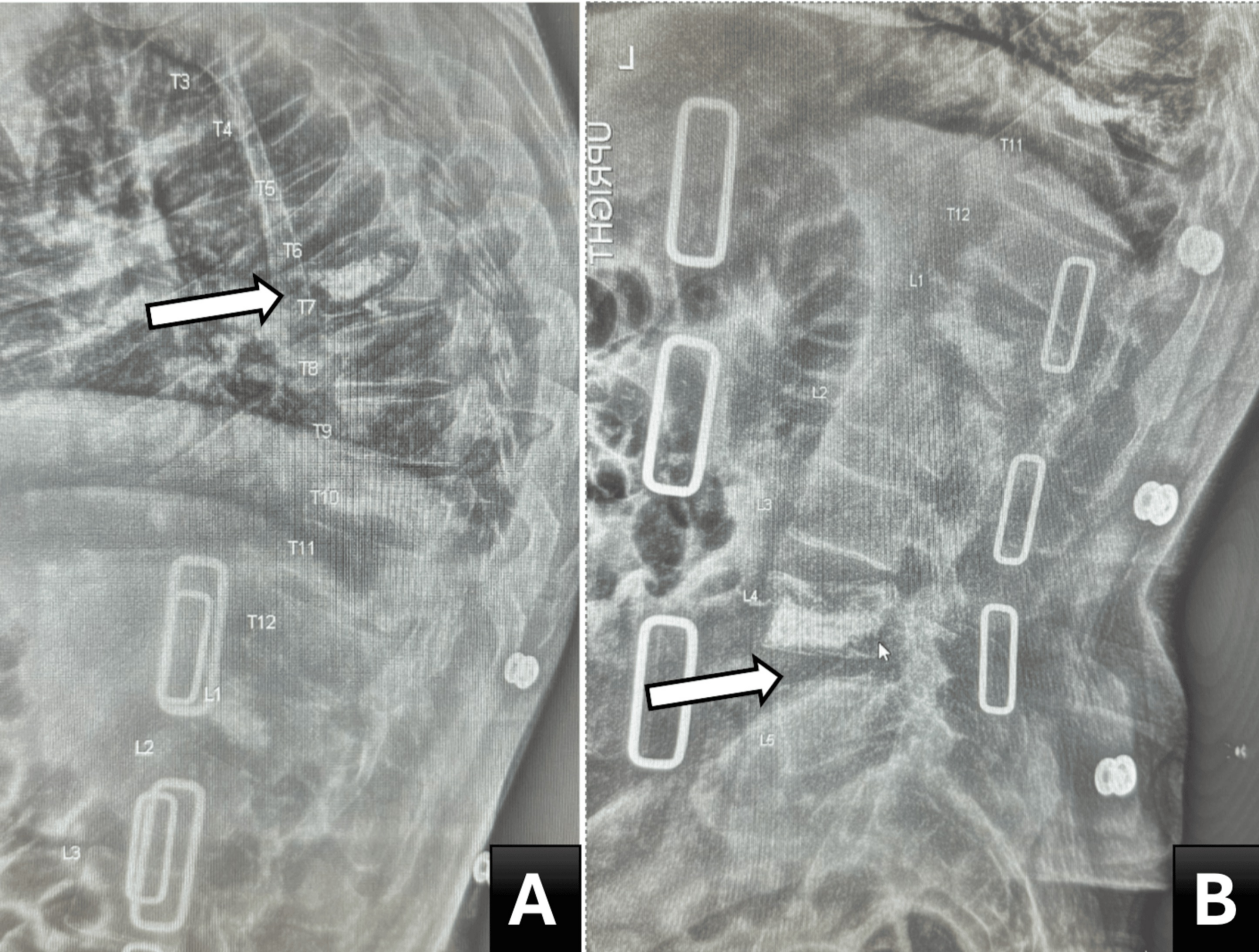
Multilevel vertebral compression deformities on plain radiography. Lateral radiographs of the thoracic (A) and lumbar (B) spine demonstrating multilevel vertebral compression deformities. Panel A highlights compression at the mid-thoracic level (arrow), while Panel B demonstrates deformity at the lower lumbar spine (arrow). Prior vertebral augmentation material is visible, consistent with previous kyphoplasty. These findings further illustrate diffuse skeletal fragility in the absence of discrete lytic lesions, supporting a systemic infiltrative process such as multiple myeloma.

Plain radiographs corroborated these findings, demonstrating multilevel vertebral compression deformities across the thoracic and lumbar spine with evidence of prior vertebral augmentation (Figure 3). Collectively, the laboratory abnormalities and multimodal imaging findings were highly suggestive of an underlying systemic plasma cell disorder, with diffuse skeletal involvement in the absence of classic focal lytic lesions, ultimately raising strong suspicion for IgA multiple myeloma.

Bone marrow examination demonstrated a markedly hypercellular marrow (approximately 70% cellularity) extensively infiltrated by atypical plasma cells. CD138 immunohistochemical staining showed plasma cells comprising approximately 90% of marrow cellularity, consistent with a markedly elevated tumor burden and advanced marrow involvement. Morphologic evaluation revealed diffuse effacement of normal hematopoietic architecture by sheets of plasma cells with suppression of erythroid, myeloid, and megakaryocytic lineages. In conjunction with anemia, thrombocytopenia, and extensive skeletal disease, these findings fulfilled International Myeloma Working Group (IMWG) diagnostic criteria for active multiple myeloma based on both CRAB features and SLiM biomarkers, including >60% clonal bone marrow plasma cells.

**Figure 4:**
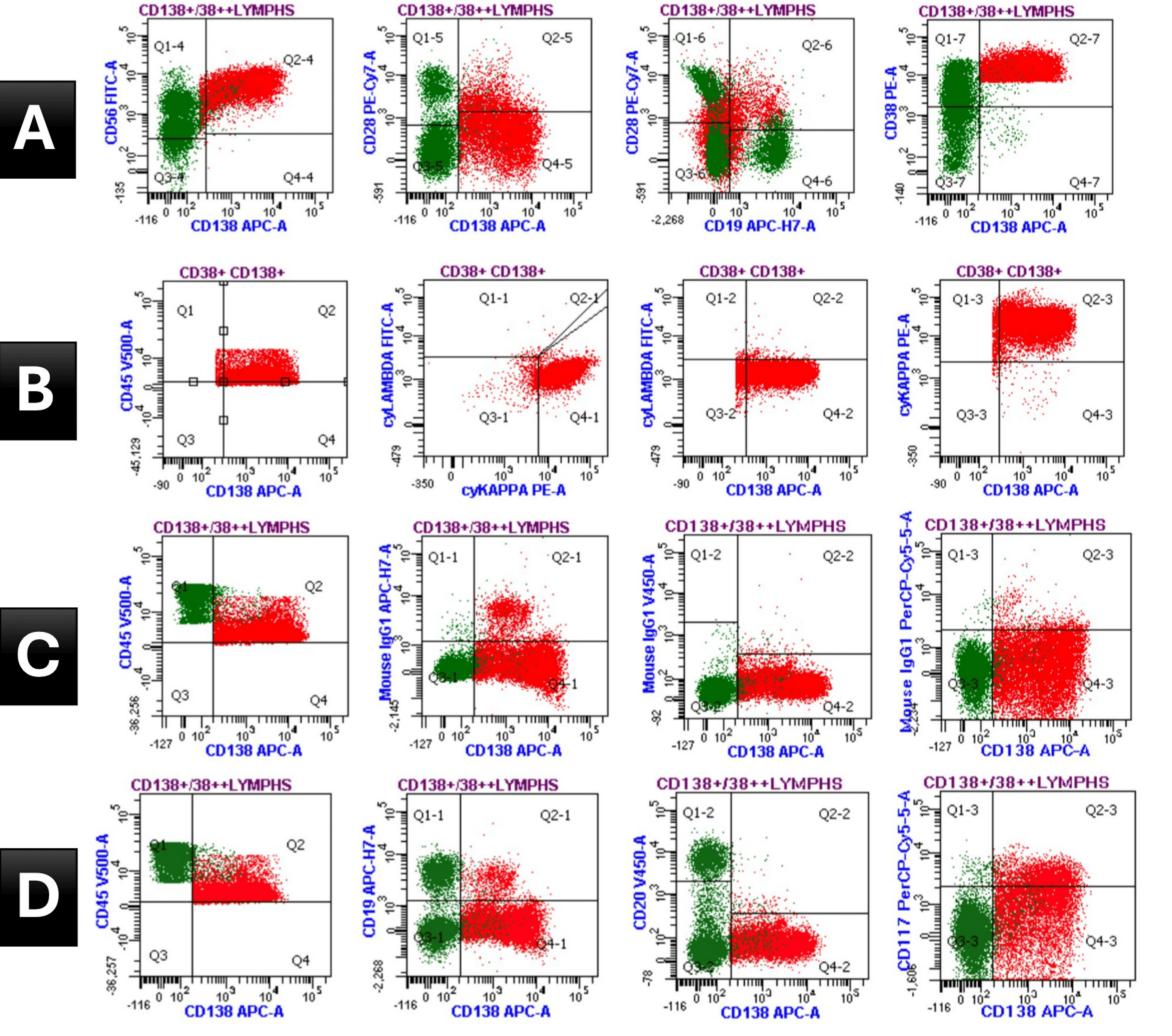
Multiparametric flow cytometric immunophenotyping of clonal plasma cells in IgA kappa multiple myeloma. Representative bone marrow flow cytometry plots demonstrating an aberrant plasma cell population (red) characterized by bright CD38 and CD138 expression with abnormal immunophenotypic features. (A) Plasma cells demonstrate uniform CD56 expression, decreased CD28 expression, loss of CD19, and retained bright CD38 positivity. (B) Plasma cells exhibit decreased CD45 expression, cytoplasmic kappa light chain restriction, and absence of cytoplasmic lambda expression, confirming clonality. (C) Additional gating plots demonstrate persistent CD138-positive plasma cells with abnormal immunoglobulin light chain expression patterns. (D) Plasma cells show loss of CD20 expression and aberrant CD117 expression. Green populations represent background lymphoid/non-plasma cell populations for comparison. Collectively, these findings support a clonal plasma cell neoplasm consistent with IgA kappa multiple myeloma.

Multiparametric flow cytometric analysis further characterized the clonal plasma cell population (Figure 4). The initial gating strategy used forward- and side-scatter characteristics to exclude debris and nonviable events, followed by identification of viable plasma cells based on bright co-expression of CD38 and CD138. Subsequent immunophenotypic analysis of the gated plasma cell population demonstrated a distinct population comprising approximately 3.4% of the total analyzed events, with cytoplasmic kappa light chain restriction and no lambda predominance, confirming clonality. The neoplastic plasma cells exhibited an aberrant immunophenotype characterized by loss of CD19 expression, decreased CD45 and CD28 expression, and aberrant expression of CD56 and CD117, while retaining strong CD38 and CD138 positivity. Additional analysis demonstrated absent CD20 expression, further supporting a malignant plasma cell phenotype. This immunophenotypic profile is characteristic of neoplastic plasma cells in multiple myeloma and strongly supports the diagnosis of IgA kappa multiple myeloma. The relatively lower plasma cell percentage detected by flow cytometry likely underestimated the true disease burden due to sampling variability and patchy marrow involvement, emphasizing the importance of correlation with core biopsy morphology and histopathologic assessment.

**Figure 5:**
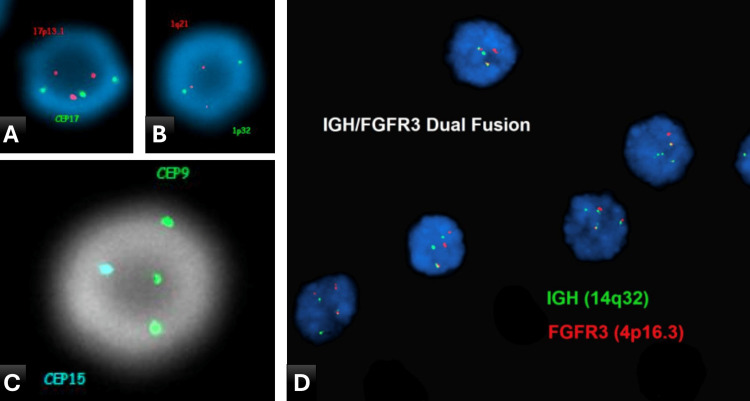
Fluorescence in situ hybridization (FISH) analysis demonstrating cytogenetic abnormalities in plasma cell myeloma. (A) Probe targeting chromosome 17 (17p13.1) showing copy number alterations (trisomy). (B) Probe targeting chromosome 1q21 demonstrating gain of 1q, a high-risk cytogenetic feature. (C) Centromeric probe for chromosome 9 (CEP9) indicating trisomy. (D) Dual fusion probe for IGH (14q32, green) and FGFR3 (4p16.3, red), illustrating copy number alterations with dual fusion. These findings are consistent with a complex cytogenetic profile associated with high-risk multiple myeloma.

Cytogenetic evaluation with fluorescence in situ hybridization (FISH) revealed multiple clinically significant abnormalities. Notably, gain of chromosome 1q was identified in a majority of analyzed cells, a well-recognized high-risk feature associated with adverse prognosis. Additional findings included trisomy 9, trisomy 17, FGFR3 gain, and immunoglobulin heavy chain (IGH) copy number alterations, reflecting a complex genomic profile characteristic of advanced plasma cell neoplasia. Representative fluorescence in situ hybridization findings demonstrating these cytogenetic abnormalities are shown in Figure 5.

Collectively, the integration of flow cytometric and cytogenetic findings provides robust immunophenotypic and molecular confirmation of a clonal plasma cell disorder, strongly supporting the diagnosis of IgA kappa multiple myeloma and highlighting high-risk disease biology.

Multidisciplinary board discussion

The case was reviewed by internal medicine, hematology, gastroenterology, orthopedic surgery, and interventional radiology. Although the patient initially presented with dark stools and severe anemia, the degree of cytopenia, marked globulin gap, elevated IgA, highly abnormal kappa/lambda ratio, and multifocal vertebral compression fractures suggested that gastrointestinal bleeding alone did not explain the full clinical picture. Hematology assessed the presentation as highly concerning for IgA multiple myeloma, with anemia and skeletal involvement as the dominant disease manifestations. Orthopedic surgery recommended TLSO bracing and outpatient spine follow-up, while interventional radiology evaluated the patient for possible kyphoplasty of the new T6 fracture and recommended further MRI characterization prior to intervention.

Tissue diagnosis

Definitive diagnosis was established through integrated immunophenotypic, laboratory, and clinicopathologic evaluation of the bone marrow. Flow cytometric analysis (Figure 4) identified a clonal plasma cell population exhibiting cytoplasmic kappa light chain restriction and an aberrant immunophenotype characterized by loss of CD19, decreased CD45 expression, and aberrant expression of CD56 and CD117, with retained CD38 and CD138 positivity. Although plasma cells accounted for approximately 3.4% of analyzed events by flow cytometry, this likely underestimated the true disease burden due to sampling variability and the patchy distribution of marrow involvement commonly seen in plasma cell neoplasms.

Peripheral blood evaluation demonstrated normocytic anemia, thrombocytopenia, and rouleaux formation, findings consistent with marrow dysfunction and elevated circulating monoclonal protein. Additional laboratory studies revealed markedly elevated IgA levels and a profoundly abnormal kappa/lambda free light chain ratio, further supporting a monoclonal plasma cell process.

Cytogenetic and fluorescence in situ hybridization (FISH) analyses (Figure 5) demonstrated a complex genomic profile, including gain of chromosome 1q, trisomy 9, trisomy 17, FGFR3 gain, and immunoglobulin heavy chain (IGH) copy number alterations, findings associated with biologically aggressive multiple myeloma and adverse prognostic risk.

In conjunction with the patient’s extensive skeletal involvement and multifocal vertebral compression fractures, these findings collectively established the diagnosis of IgA kappa multiple myeloma with high-risk cytogenetic features and advanced systemic disease burden.

Management

The patient received packed red blood cell transfusions for severe symptomatic anemia, with improvement in hemoglobin from approximately 5.4 g/dL to the 9 g/dL range. He was treated with proton pump inhibitor therapy for severe esophagitis and esophageal ulceration and was advised to avoid aspirin, ibuprofen, naproxen, and other nonsteroidal anti-inflammatory drugs. Pain control was provided for vertebral compression fractures, and the patient was maintained in a TLSO brace with instructions to wear it when out of bed. After hematology evaluation raised strong concern for IgA multiple myeloma, dexamethasone 40 mg daily was initiated, with plans to complete a four-day course and transition to outpatient hematology follow-up for definitive treatment planning after completion of marrow studies. 

Clinical outcome

The patient remained clinically stable during hospitalization following transfusion support, with no evidence of active infection or neurologic compromise related to the acute T6 compression fracture. Gastrointestinal evaluation did not demonstrate active bleeding at the time of endoscopy, although severe esophagitis and an esophageal ulcer were identified. His anemia improved after transfusion; however, thrombocytopenia, monoclonal protein abnormalities, and extensive skeletal disease persisted. Although the clinical suspicion for multiple myeloma was high during admission, definitive anti-myeloma therapy was deferred pending completion of bone marrow biopsy, flow cytometric, and cytogenetic characterization to establish disease subtype and risk stratification prior to treatment initiation. Following confirmation of IgA kappa multiple myeloma, the patient was discharged home in stable condition, with urgent outpatient hematology follow-up arranged to initiate definitive therapy, in addition to follow-up with gastroenterology, interventional radiology, and orthopedic spine services.

## Discussion

Background (history, epidemiology, and WHO classification) 

Multiple myeloma (MM) is a malignant plasma cell neoplasm first described in the 19th century, historically characterized by bone pain, anemia, and abnormal serum proteins [6]. It is now recognized as a biologically heterogeneous disease defined by clonal plasma cell proliferation within the bone marrow and associated end-organ damage, classically summarized by the CRAB criteria (hypercalcemia, renal dysfunction, anemia, and bone lesions) [6]. MM accounts for approximately 10% of hematologic malignancies and remains the second most common blood cancer worldwide [7,8].

The incidence of MM increases with age, with a median age at diagnosis of approximately 65-70 years, and demonstrates a higher prevalence in males and certain ethnic populations [7,9]. Established risk factors include advanced age, male sex, obesity, environmental exposures, and precursor plasma cell disorders such as monoclonal gammopathy of undetermined significance (MGUS), which universally precedes symptomatic disease [1,10]. 

MM primarily involves the bone marrow, with malignant plasma cells promoting osteoclast activation and suppressing osteoblast function, resulting in lytic lesions and skeletal fragility that most commonly affect the axial skeleton [11]. According to the World Health Organization classification, MM is categorized among plasma cell neoplasms, with subtypes defined by immunoglobulin expression, including IgG and IgA variants [4]. IgA myeloma, accounting for approximately 20% of cases, is often associated with higher tumor burden and more atypical clinical presentations, which may complicate early recognition [12]. Despite advances in therapy, MM remains incurable, and delays in diagnosis, particularly in cases lacking classic CRAB features, continue to impact outcomes [1,4,9].

Pathogenesis and pathophysiology

Multiple myeloma arises from the malignant transformation of post-germinal center plasma cells that clonally expand within the bone marrow and produce monoclonal immunoglobulins [4,9]. The disease is initiated by primary cytogenetic events, most commonly immunoglobulin heavy chain (IgH) translocations involving chromosome 14q32, followed by secondary mutations affecting key oncogenic pathways, including RAS, NF-κB, and MYC, which drive proliferation and survival [4,13].

Disease progression is sustained by interactions between malignant plasma cells and the bone marrow microenvironment, where cytokines such as interleukin-6 promote tumor growth and resistance to apoptosis [4]. Myeloma cells disrupt normal bone remodeling by increasing osteoclast activity and suppressing osteoblast function, resulting in osteolytic lesions and skeletal fragility [11].

Marrow infiltration by clonal plasma cells impairs normal hematopoiesis, leading to anemia and other cytopenias [1]. Concurrently, excessive production of monoclonal immunoglobulins contributes to hyperproteinemia and the globulin gap observed in this patient [4]. Histopathologically, multiple myeloma is characterized by clonal plasma cell infiltration with immunophenotypic restriction, confirming a monoclonal process [1].

Comparative analysis of our case with the existing literature

Clinical Presentation

Multiple myeloma (MM) classically presents with bone pain, anemia, renal dysfunction, hypercalcemia, and radiographic evidence of osteolytic lesions, with diagnosis supported by clonal plasma cell proliferation and myeloma-defining events such as CRAB features or biomarker criteria, including an abnormal free light chain ratio [1]. In contrast, our patient presented with severe symptomatic anemia in the setting of recurrent gastrointestinal bleeding, a clinical picture that strongly suggested an isolated gastrointestinal etiology. The absence of renal dysfunction and hypercalcemia further obscured the diagnosis and delayed consideration of an underlying plasma cell disorder. Similar diagnostic challenges have been described in rare reports where gastrointestinal bleeding preceded the recognition of MM, often leading to anchoring bias and delayed workup for hematologic malignancy [14]. However, unlike cases involving direct gastrointestinal involvement by plasmacytoma, where bleeding results from mucosal infiltration by malignant plasma cells [15], our patient had no evidence of gastrointestinal tumor involvement on endoscopy. This distinction underscores a unique diagnostic pathway in which a competing, plausible diagnosis masked the underlying malignancy.

Clinical Laboratory

IgA multiple myeloma has been reported to exhibit a distinct clinical and laboratory profile compared with other immunoglobulin subtypes, frequently presenting with anemia, elevated monoclonal protein burden, extensive bone disease, and more aggressive clinical behavior [3,12]. Wang et al. reported that patients with IgA myeloma often demonstrate higher tumor burden and less readily detectable monoclonal spikes on routine electrophoresis, which may contribute to delayed recognition and diagnostic complexity [3]. Similarly, IgA myeloma has been associated with increased serum viscosity, advanced marrow infiltration, and poorer prognostic characteristics compared with non-IgA variants [12].

Our patient shared several characteristic features of IgA multiple myeloma, including severe anemia, marked hyperproteinemia with a pronounced globulin gap, substantially elevated IgA levels, kappa-restricted monoclonal disease, and extensive skeletal involvement [1,3]. However, the presentation also differed from more classic manifestations in several clinically important respects. Despite advanced disease burden, renal function remained preserved, and serum calcium levels were normal, thereby lacking two traditional CRAB features commonly associated with symptomatic multiple myeloma [1]. In addition, imaging demonstrated diffuse osteopenia and recurrent vertebral compression fractures without discrete osteolytic lesions, representing a less recognized radiographic phenotype of myeloma-related skeletal disease [11,16].

This atypical presentation created a substantial diagnostic challenge, as the patient’s severe anemia and dark stools initially suggested recurrent gastrointestinal bleeding as the primary etiology. Similar diagnostic delays have been described in reports where clinically plausible alternative diagnoses obscured underlying plasma cell dyscrasia [14,15]. In this case, however, the persistent globulin gap, cytopenias, abnormal free light chain ratio, and progressive skeletal fragility ultimately redirected the diagnostic evaluation toward plasma cell malignancy. Collectively, this case reinforces that unexplained hyperproteinemia, elevated globulin gap, and diffuse skeletal fragility should prompt evaluation for plasma cell dyscrasia even in the absence of classic lytic lesions, renal dysfunction, or hypercalcemia [1,4,11].

Diagnostic Workup

The diagnostic evaluation in this case highlights the critical role of integrated laboratory, morphologic, immunophenotypic, and cytogenetic assessment in establishing the diagnosis of multiple myeloma (MM) [1,4]. While serum studies initially raised suspicion for plasma cell dyscrasia through marked hyperproteinemia, a pronounced globulin gap, markedly elevated IgA levels, and a profoundly abnormal kappa/lambda free light chain ratio, definitive diagnosis required bone marrow characterization and molecular profiling [1,3,4].

Bone marrow examination demonstrated a markedly hypercellular marrow with extensive plasma cell infiltration comprising approximately 90% of marrow cellularity by CD138 immunohistochemical staining. Morphologic evaluation revealed diffuse replacement of normal hematopoietic architecture by sheets of atypical plasma cells with associated suppression of erythroid, myeloid, and megakaryocytic lineages, explaining the patient’s persistent cytopenias [1]. These findings fulfilled International Myeloma Working Group (IMWG) diagnostic criteria for active MM through both CRAB features and SLiM biomarkers, including >60% clonal bone marrow plasma cells [1].

Multiparametric flow cytometry further characterized the neoplastic plasma cell population and provided important immunophenotypic confirmation of clonality. The abnormal plasma cells demonstrated bright CD38 and CD138 expression with cytoplasmic kappa light chain restriction and absence of lambda predominance, confirming a monoclonal plasma cell process. Additional aberrancies included loss of CD19 expression, decreased CD45 and CD28 expression, and aberrant expression of CD56 and CD117, a phenotype characteristic of malignant plasma cells in MM [13]. These immunophenotypic findings are clinically important because loss of normal B-cell markers and aberrant adhesion molecule expression help distinguish neoplastic plasma cells from reactive plasma cell proliferations and may correlate with disease biology, marrow homing behavior, and disease progression [13].

Cytogenetic and fluorescence in situ hybridization (FISH) analyses further demonstrated a complex genomic profile, including gain of chromosome 1q, trisomies involving chromosomes 9 and 17, FGFR3 abnormalities, and IGH alterations. These molecular findings are particularly relevant in IgA myeloma, as high-risk cytogenetic abnormalities such as 1q gain and FGFR3/IGH-associated abnormalities have been associated with aggressive disease biology, treatment resistance, and inferior survival outcomes [4,13]. Complex hyperdiploidy and secondary chromosomal abnormalities are increasingly recognized as major determinants of biologic heterogeneity and prognosis in MM [4]. The coexistence of extensive marrow infiltration with high-risk molecular abnormalities in our patient likely contributed to the substantial skeletal disease burden observed at presentation [4,13].

Skeletal involvement in MM is well established and results from increased osteoclast activity and suppressed osteoblast function, leading to bone resorption and structural fragility [11,16]. Our patient demonstrated extensive vertebral compression fractures initially attributed to degenerative disease, a pattern consistent with prior literature describing underrecognized myeloma-related skeletal pathology, particularly in younger patients or those without classic imaging findings [16]. Notably, despite extensive skeletal involvement, no discrete osteolytic lesions were identified on serial imaging. This highlights a less recognized radiographic phenotype of MM characterized by diffuse osteopenia and marrow infiltration rather than focal osteolysis, which may contribute to misdiagnosis as osteoporosis or degenerative spine disease [11,16]. The presence of recurrent fractures despite prior kyphoplasty further supports a systemic and progressive underlying process rather than isolated orthopedic pathology.

Management

Management in this case was consistent with current standards of care for newly suspected MM. Initial stabilization with transfusion support for severe anemia and corticosteroid therapy with dexamethasone aligns with guideline-supported approaches for symptomatic disease and rapid cytoreduction [1,17]. Definitive treatment planning with combination systemic therapy remains the cornerstone of MM management, pending cytogenetic and molecular characterization.

In addition, supportive and multidisciplinary interventions were appropriately implemented. Orthopedic management with TLSO bracing and evaluation for vertebral augmentation reflects recommended strategies for managing myeloma-related bone disease and preventing further skeletal complications [11]. Gastroenterologic management addressed coexisting esophagitis and ulcer disease, while interventional radiology contributed to procedural planning. This coordinated, multidisciplinary approach is consistent with best practices in MM care and highlights the importance of integrating multiple specialties when clinical presentation is complex and multifactorial.

Outcome and Follow-up

Prognosis in MM has improved significantly with advances in targeted and combination therapies; however, outcomes remain closely linked to disease burden, cytogenetic risk, and response to treatment [1]. In this patient, preserved renal function represents a favorable prognostic factor, while severe anemia, thrombocytopenia, and markedly elevated IgA levels suggest substantial marrow involvement and high disease burden at presentation.

Compared with previously reported cases, the distinguishing feature of this case lies not in an unusual site of involvement but in the diagnostic challenge posed by a competing and clinically plausible alternative diagnosis. The delay in recognizing MM was not due to the absence of diagnostic criteria but rather due to the attribution of symptoms to gastrointestinal pathology. This highlights a critical clinical lesson: biochemical abnormalities such as hyperproteinemia and the globulin gap may serve as early and decisive indicators of plasma cell dyscrasia, even when the clinical presentation appears to support a different primary diagnosis.

What have we learned from this case

This case highlights an uncommon and diagnostically deceptive presentation of IgA multiple myeloma in which the disease failed to conform to the classic clinical expectations traditionally associated with symptomatic myeloma. Rather than presenting with the typical constellation of CRAB findings, the patient demonstrated a more subtle but clinically progressive pattern characterized by profound hyperproteinemia, persistent cytopenias, diffuse skeletal fragility, and recurrent vertebral compression fractures in the absence of discrete osteolytic lesions, renal dysfunction, or hypercalcemia. The case, therefore, illustrates how strict reliance on conventional diagnostic patterns may delay recognition of clinically significant plasma cell disease.

The most distinctive feature of this presentation was the marked discordance between the severity of skeletal involvement and the absence of focal lytic lesions on serial imaging. Instead of classic punched-out lesions, the patient exhibited diffuse osteopenia with recurrent multilevel compression fractures despite prior kyphoplasty, suggesting a diffuse infiltrative skeletal process rather than localized cortical destruction. This radiographic phenotype can easily be misinterpreted as osteoporosis, degenerative spine disease, or age-related skeletal fragility, particularly when competing clinical diagnoses are present.

Equally important, this case demonstrates the diagnostic value of biochemical abnormalities that may initially appear nonspecific. In this patient, the persistent globulin gap and marked hyperproteinemia ultimately became the pivotal clues that redirected the workup toward plasma cell dyscrasia. In retrospect, these laboratory abnormalities were more diagnostically informative than the imaging findings themselves and proved essential in identifying the underlying malignancy.

Another important lesson from this case is the degree to which anchoring bias can influence diagnostic reasoning. The coexistence of recurrent gastrointestinal bleeding and chronic vertebral disease created a clinically plausible alternative explanation for the patient’s symptoms, delaying broader hematologic evaluation despite progressive systemic findings. This emphasizes the importance of reassessing the diagnostic framework when laboratory abnormalities, cytopenias, or skeletal progression become disproportionate to the presumed primary diagnosis.

Finally, this case underscores the importance of integrating advanced hematopathologic and molecular diagnostics into modern myeloma evaluation. Comprehensive bone marrow morphology, multiparametric flow cytometry, and cytogenetic profiling not only confirmed the diagnosis but also revealed biologically aggressive disease despite the atypical clinical presentation. Collectively, this case expands the recognized phenotypic spectrum of IgA multiple myeloma and reinforces the need for heightened clinical suspicion when diffuse skeletal fragility and unexplained biochemical abnormalities coexist, even in the absence of classic radiographic or metabolic manifestations.

## Conclusions

This case illustrates an atypical presentation of IgA multiple myeloma in which recurrent gastrointestinal bleeding and severe anemia obscured the underlying diagnosis. The presence of persistent cytopenias and a marked globulin gap ultimately served as the critical diagnostic pivot, emphasizing the importance of recognizing hyperproteinemia as an early clue to plasma cell dyscrasia. This report highlights how multiple myeloma may present without classic CRAB features and instead manifest through competing clinical conditions, leading to potential diagnostic delay. The key insight is the diagnostic value of integrating routine laboratory abnormalities, particularly the globulin gap, into clinical reasoning when the presentation is incongruent with a single etiology. For clinicians, this underscores the need to maintain a high index of suspicion for hematologic malignancy in patients with unexplained anemia and abnormal protein profiles. Future work should further evaluate the role of early biochemical markers in improving the timely detection of atypical multiple myeloma presentations.
